# Analysis of Metabolic Differences and Core Regulatory Pathways in Lactic Acid Bacteria-Fermented Broths of Different *Ziziphus jujuba* Mill. Varieties Based on LC-MS Untargeted Metabolomics

**DOI:** 10.3390/foods15061071

**Published:** 2026-03-18

**Authors:** Jiangning Zhang, Zheng Ye

**Affiliations:** Shanxi Institute for Functional Food, Shanxi Agricultural University, Taiyuan 030001, China; 13613467239@163.com

**Keywords:** untargeted metabolomics, LC-MS, *Ziziphus jujuba* Mill., lactic acid bacteria fermentation, differential metabolites, metabolic regulatory pathways

## Abstract

*Ziziphus jujuba* Mill. is a characteristic resource with both medicinal and edible values. At present, its lactic acid bacteria-fermented products are plagued by ambiguous variety selection and low added value. To clarify the variety-specific regulatory effects of *Z. jujuba* cultivars on metabolic profiles during lactic acid bacteria fermentation, this study analyzed the metabolic characteristics of fermented broths of Tan jujube, Jun jujube, and Ban jujube under a unified fermentation system using LC-MS untargeted metabolomics technology. Significantly differential metabolites were screened with the criteria of *p* < 0.05 and VIP > 1, and the metabolic regulatory mechanisms were further elucidated, combined with KEGG pathway enrichment analysis. The results showed that a total of 570 metabolites were identified in the three fermented broths. Tan jujube was enriched in linolenic acid, Ban jujube was rich in D-xylitol and dethiobiotin, and Jun jujube had prominent contents of S-adenosylmethionine and pyridoxine. All the aforementioned metabolites are involved in important physiological processes such as anti-inflammation and intestinal homeostasis maintenance. The differential metabolites were mainly enriched in 6 key pathways, including central carbon metabolism, ABC transporters, and phenylpropanoid biosynthesis, among which central carbon metabolism and ABC transporters were the core regulatory pathways. This study constructed an association network of *Z. jujuba* variety–differential metabolite–key pathway, systematically elucidated the metabolic differentiation mechanisms of fermented broths from different *Z. jujuba* cultivars, and provided a scientific basis for the precise selection of *Z. jujuba* varieties dedicated to fermentation and the targeted development of high-value-added functional fermented foods.

## 1. Introduction

*Ziziphus jujuba* Mill., an important fruit species with both medicinal and edible properties, belongs to the family Rhamnaceae [1]. It is a deciduous shrub or small tree characterized by diverse morphological traits and a well-developed root system. Its fruit, rich in nutritional and bioactive compounds, has been utilized for centuries in traditional medicine and food systems, underscoring its ethnobotanical significance. Statistics indicate that China boasts extremely abundant *Z. jujuba* resources [2], among which Tan jujube, Jun jujube, and Ban jujube are the most extensively cultivated varieties [3]. However, the current deep-processed *Z. jujuba* products are still confronted with problems such as a single variety, low added value, and inadequate development of functional foods [4,5]. As an efficient biotransformation approach [6], lactic acid bacteria fermentation can significantly enhance the flavor, nutritional characteristics, and biological activity of *Z. jujuba* [7,8], which serves as an effective way to develop high-value-added functional *Z. jujuba* products. Therefore, an in-depth analysis of the metabolic differences among different *Z. jujuba* varieties during fermentation by means of modern metabolomics technology [9] is of great practical significance for developing high-quality and diversified functional *Z. jujuba* products and increasing industrial added value.

In recent years, metabolomics technology has been widely applied in the field of food science [10,11,12,13]. Researchers have clarified the mechanism by which *Rosa xanthina* fruits delay aging through regulating oxidative stress and promoting collagen synthesis based on NMR metabolomics [14]; others have explored the potential effects of lead stress on metabolic pathways and dynamic changes in metabolites of barley by combining LC-MS metabolomics with principal component analysis (PCA) [15]. These studies have demonstrated that metabolomic analysis enables comprehensive qualitative and quantitative analysis of small-molecule metabolites under specific conditions and plays a crucial role in elucidating the structural composition and bioavailability mechanisms of molecular substances.

Although numerous studies have been conducted on the metabolites of *Ziziphus jujuba* Mill., most of them only focus on the analysis of a single class of metabolites (e.g., polysaccharides [16,17], cyclic nucleotides [18,19]), and there is a lack of pairwise comparative studies on fermented broths of different *Z. jujuba* varieties under a unified fermentation system [20,21,22]. Therefore, this study intends to adopt LC-MS untargeted metabolomics technology to conduct a systematic pairwise comparative analysis on the fermented broths of Tan jujube, Jun jujube, and Ban jujube after lactic acid bacteria fermentation. By means of cluster heatmap, sample hierarchical clustering tree, volcano plot, differential metabolite Z-score plot, and KEGG pathway enrichment analysis, this study aims to reveal the metabolic fingerprint differences among fermented broths of different *Z. jujuba* varieties, screen key differential metabolites, and elucidate their enriched pathways. The results of this study can not only provide a theoretical basis for the in-depth development of jujube fermented products but also offer a scientific reference for the research, development, and application of functional jujube fermented products.

## 2. Materials and Methods

### 2.1. Materials, Reagents, and Instruments

#### 2.1.1. Materials and Reagents

Jujube raw materials: Liulin Tan jujube, Jiaocheng Jun jujube, and Jishan Ban jujube, all provided by Shanxi Tianzi Industrial Co., Ltd. (Taiyuan, Shanxi, China). The fresh fruits were collected from orchards in Liulin (37°27′ N, 110°57′ E), Jiaocheng (37°33′ N, 111°14′ E), and Jishan (35°35′ N, 110°58′ E), Shanxi Province, China, on October 15, 2023. These varieties are well-characterized geographical indication products, and their identities were verified based on the official descriptions provided in the geographical indication registrations [23,24,25] and the Flora of China [26]. Composite lactic acid bacteria were purchased from Zhongke Jiayi Bioengineering Co., Ltd. (Weifang, China), which consisted of five strains: *Lactobacillus rhamnosus* PB-LR76 (24.7%), *Limosilactobacillus reuteri* PB-LR09 (6.2%), *Lactiplantibacillus plantarum* HH-LP56 (39.5%), *Bifidobacterium animalis subsp. Lactis* HH-BA68 (24.7%) and *Lactobacillus acidophilus* HH-LA26 (4.9%). MRS broth (Lactobacillus de Man, Rogosa, and Sharpe broth) was used as the activation medium. Acetonitrile (chromatographic grade) was purchased from Thermo Fisher Scientific (Waltham, MA, USA); formic acid (chromatographic grade) was purchased from TCI (Tokyo, Japan); ammonium formate was purchased from Sigma-Aldrich (St. Louis, MO, USA); BE-2600 filter membranes were purchased from Tianjin Jinteng Technology Co., Ltd. (Tianjin, China).

#### 2.1.2. Instruments

We used a 5305 vacuum concentrator (Eppendorf, Hamburg, Germany); TGL-16GB high-speed desktop centrifuge (Jintan Dadi Automation Instrument Factory, Jintan, China); Vanquish high-performance liquid chromatography (HPLC) system (Thermo Fisher Scientific, Waltham, MA, USA); and Q Exactive Focus mass spectrometer (Thermo Fisher Scientific, Waltham, MA, USA).

### 2.2. Preparation of Fermented Jujube Broths

Seventy grams each of the three jujube varieties (Tan jujube, Jun jujube, and Ban jujube), after cleaning and stoning, were weighed and boiled with approximately 500 mL of distilled water. The soluble solid content was monitored in real time during boiling until it reached 22 °Brix. After boiling, the residue was removed by filtration, and the soluble solid content of the filtrate was accurately determined to be 22 °Brix using a refractometer. The filtrate was then cooled to obtain the raw jujube juice.

The composite lactic acid bacteria were stored at −20 °C. Prior to use, the bacteria were activated twice (24 h for each activation) in sterilized MRS broth at 37 °C to ensure high viability and activity, and the activated bacterial suspension was used as the inoculum for fermentation experiments. Ten milliliters of the raw juice of each jujube variety was inoculated with the composite lactic acid bacteria starter at an inoculum size of 0.5% (*v*/*v*), with the fermentation temperature, time, and inoculum size determined based on the results of preliminary pre-experiments. The inoculated juice was cultured in a fermenter at 37 °C for 100 h to obtain the fermented jujube broths for subsequent analysis, which were then stored at −20 °C [27].

Three jujube varieties were included in this study: Tan jujube, Jun jujube, and Ban jujube. To ensure the accuracy and scientific validity of the analytical results, three biological replicates were prepared for each variety. Specifically, samples for each variety were collected from three different trees within the same orchard to represent natural biological variability. The three replicate samples of the same variety were grouped together and numbered as one (Tan jujube), two (Jun jujube), and three (Ban jujube), respectively. A pairwise comparative analysis was subsequently performed on the three sample groups to investigate the differences in their differential metabolites.

### 2.3. Metabolite Extraction

Fresh samples were immediately frozen at −80 °C for 24 h and then subjected to freeze-drying. The experimental samples were thawed at 4 °C and vortexed for 1 min to achieve homogeneous mixing after thawing. Methanol solution (stored at −20 °C, 500 µL) was added to the dried sample tubes, followed by another 1 min of vortexing. The mixture was then centrifuged at 12,000 r/min for 10 min at 4 °C. The entire supernatant was transferred to new 2 mL centrifuge tubes and subjected to vacuum concentration and drying. The dried residues were accurately reconstituted with 150 µL of 2-chloro-L-phenylalanine solution (0.004 mg/mL, prepared in 80% methanol-water, stored at −20 °C). The supernatant was collected and filtered through a 0.22 µm filter membrane, and the resulting filtrate was transferred to dedicated vials [28]. The vials were then stored in the autosampler at 4 °C until LC-MS analysis.

### 2.4. LC-MS Detection

Metabolomic analysis of the three fermented jujube broths was performed by coupling a Thermo Vanquish ultra-high-performance liquid chromatography (UHPLC) system with a Thermo Q Exactive Focus mass spectrometer [29,30,31].

UHPLC conditions: The UHPLC system was equipped with an ACQUITY UPLC^®^ HSS T3 column (2.1 mm × 150 mm, 1.8 µm; Waters, Milford, MA, USA); the flow rate was set at 0.25 mL/min; the column temperature was maintained at 40 °C; and the injection volume was 2 μL. The sample compartment temperature was maintained at 4 °C.

Positive ion mode: The mobile phase consisted of 0.1% formic acid in acetonitrile (Phase C) and 0.1% formic acid in water (Phase D). The gradient elution program was set as follows: 0–1 min, 2% C; 1–9 min, 2–50% C; 9–12 min, 50–98% C; 12–13.5 min, 98% C; 13.5–14 min, 98–2% C; 14–20 min, 2% C. The column was re-equilibrated for 6 min before the next injection.

Negative ion mode: The mobile phase consisted of acetonitrile (Phase A) and 5 mM ammonium formate aqueous solution (Phase B). The gradient elution program was set as follows: 0–1 min, 2% A; 1–9 min, 2–50% A; 9–12 min, 50–98% A; 12–13.5 min, 98% A; 13.5–14 min, 98–2% A; 14–17 min, 2% A. The column was re-equilibrated for 3 min before the next injection.

Mass spectrometry (MS) conditions: An electrospray ionization (ESI) source was employed, with data acquired in both positive and negative ion modes separately. Voltage parameters: The spray voltage was 3.50 kV for positive ion mode and −2.50 kV for negative ion mode. Gas parameters: Sheath gas flow was 30 arb, and auxiliary gas flow was 10 arb. Temperature parameter: The capillary temperature was set at 325 °C. Scanning parameters: Full MS1 scan was conducted at a resolution of 70,000, with the MS1 scan range of m/z 81~1000. MS2 fragmentation: Higher-energy collisional dissociation (HCD) was used for MS2 fragmentation with a collision energy of 30%, and the MS2 resolution was 17,500. Acquisition mode: Data-Dependent Acquisition (DDA) was adopted, in which the top 3 precursor ions with the strongest signals were selected for fragmentation. Dynamic exclusion: Dynamic exclusion was simultaneously applied to eliminate redundant MS/MS information.

### 2.5. Data Processing and Analysis

Data preprocessing was performed using the ProteoWizard software package (v3.0). Data correction was conducted via the LOESS signal correction method based on quality control (QC) samples to eliminate systematic errors. In the data quality control step, metabolites with a relative standard deviation (RSD) > 30% in QC samples were filtered out [32].

*p*-values were calculated by statistical tests, and variable importance in projection (VIP) values were obtained via the orthogonal partial least squares–discriminant analysis (OPLS-DA) dimensionality reduction method [33]. The OPLS-DA models for all group comparisons demonstrated excellent fitness and predictive ability, with R^2^Y values of 1 and Q^2^ values ranging from 0.953 to 0.979, indicating that the models were not overfitted. Fold change was also calculated to measure the fold difference in metabolite levels among groups. The above indicators were used to evaluate the influence intensity and interpretability of each metabolite content on sample classification and discrimination, thereby assisting in the screening of differential metabolites. The screening criteria were set as follows: a metabolite was considered to have statistical significance when its *p*-value < 0.05 and VIP value > 1. Although False Discovery Rate (FDR) correction is commonly applied in untargeted metabolomics, we relied on the combined threshold of VIP > 1 and a stringent *p*-value threshold (*p* < 0.005) in this study. This approach integrates multivariate statistical analysis with a rigorous univariate threshold, effectively minimizing the risk of false positives and ensuring the reliability of the identified differential metabolites.

For pathway analysis, enrichment analysis of differential metabolites was carried out using the MetaboAnalyst software package (v4.0) [34], and the KEGG-Mapper visualization tool was employed to map and plot the correlations between differential metabolites and metabolic pathways.

## 3. Results

### 3.1. Analysis of Global Metabolites

#### 3.1.1. Cluster Heatmap of Global Metabolites

**Figure 1:**
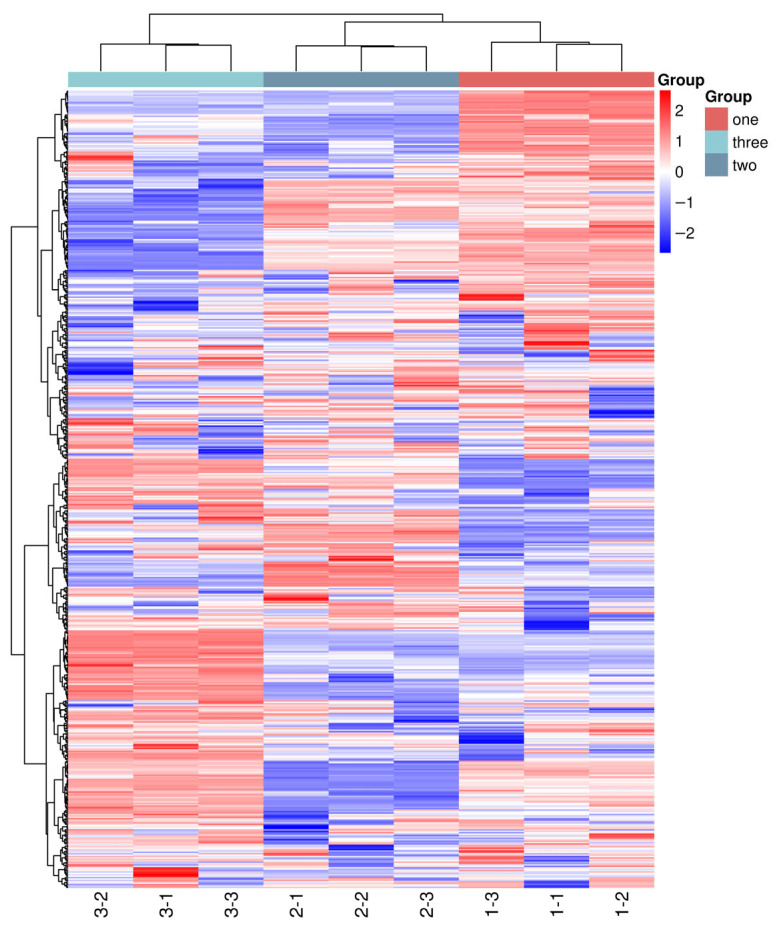
Cluster heatmap of global metabolites. Note: Red = up-regulated metabolites, Blue = down-regulated metabolites, VIP > 1 and *p* < 0.05.

Based on the metabolite data of nine samples, a cluster heatmap of global metabolites was plotted (Figure 1). This heatmap displayed the relative abundances of a total of 570 metabolites, with red indicating high expression levels and blue representing low expression levels. Through visualization, the cluster heatmap clearly revealed the metabolic differences among different samples; it could effectively retain metabolites with significant differences and also highlight those with minor differences [35,36].

As shown in Figure 1, the fermented broths of the three jujube varieties (one, two, three) contained a diverse array of metabolites, and each exhibited distinct metabolic characteristics. Specifically, group one (Tan jujube) exhibited a relatively higher abundance of amino acids and their derivatives, such as L-glutamic acid, L-isoleucine, and L-arginine, as well as nitrogen-containing compounds like carnosine. In contrast, group three (Ban jujube) was characterized by significantly elevated levels of phenolic acids (e.g., protocatechuic acid, vanillic acid, and ferulic acid) and flavonoids (e.g., quercetin, luteolin, and kaempferol). Group two (Jun jujube) showed a more balanced profile with intermediate levels of both categories. Obvious distinctions were observed in the metabolite expression patterns among the different groups (one, two, three), indicating that significant differences existed in the metabolic pathways of different jujube varieties during fermentation.

KEGG pathway enrichment analysis revealed that these differentially accumulated metabolites were primarily involved in amino acid biosynthesis, phenylpropanoid biosynthesis, flavonoid biosynthesis, and the citrate cycle (TCA cycle) pathways. The three parallel samples within each group showed high similarity with consistent color patterns, demonstrating good intragroup repeatability. Some metabolites displayed extremely high or low expression in specific groups: certain metabolites in one group were shown in red, while the corresponding positions in the other group were in blue, which served as potential biomarker candidates for distinguishing the fermented broths of different varieties, providing a visual basis for subsequent quantitative screening. This result fully demonstrated that the fermented broths of different jujube varieties possessed unique metabolic fingerprints, which provided important clues for the subsequent screening and identification of differential metabolites [37].

#### 3.1.2. Hierarchical Clustering Tree of Global Samples

**Figure 2:**
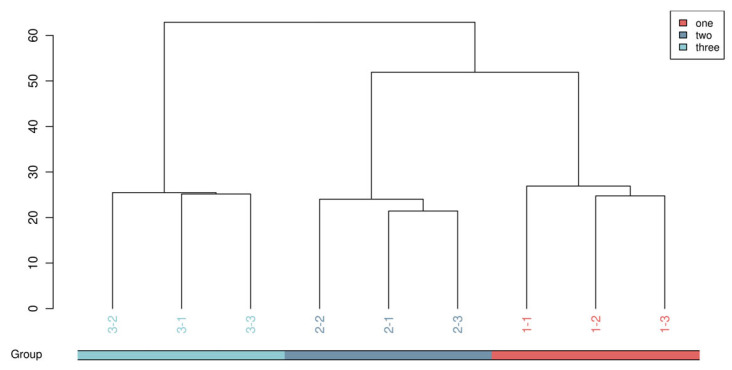
Hierarchical clustering analysis of the samples based on the metabolite profiles.

A hierarchical clustering tree was constructed (Figure 2) by processing the metabolite data of the nine samples with mathematical models. As shown in Figure 2, the clustering results were distinct and well-classified, indicating significant inter-group differences in metabolites among the three sample groups (one, two, three), while the intra-group samples exhibited high similarity and good clustering performance [38]. This result provided a reliable foundation for the subsequent screening and identification of differential metabolites.

The three main branches corresponded to the one, two, and three groups, respectively, with clear boundaries between each other, which demonstrated a significant difference in metabolite composition among groups. The three parallel samples in each group were closely clustered together, verifying the repeatability and reliability of the experiment. The structure of the clustering tree also indicated the degree of metabolic similarity among the fermented broths of different jujube varieties, providing an intuitive perspective for understanding their metabolic differences.

### 3.2. Analysis of Differential Metabolites

**Figure 3:**
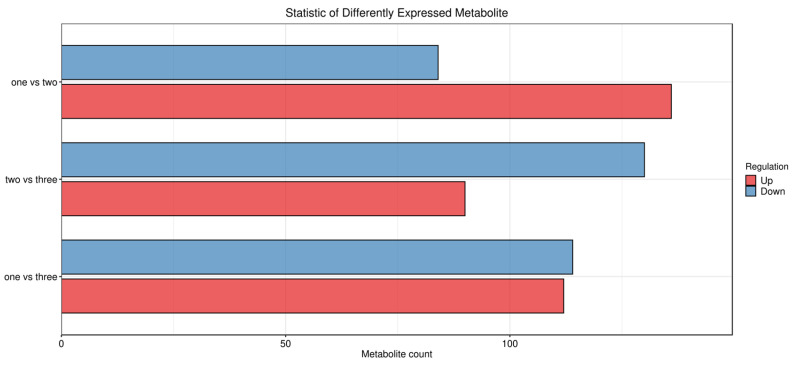
Statistical Histogram of Differential Metabolites in Intergroup Comparisons.

Differential metabolites in the sample data were screened according to the preset criteria (*p*-value < 0.05 and VIP value > 1), with the screening results presented as a histogram (Figure 3). As shown in Figure 3, abundant differential metabolites were identified in the three comparison groups (one vs. two, one vs. three, two vs. three): 82 metabolites in one vs. two, 78 in one vs. three, and 91 in two vs. three, all exceeding 75 species. This indicated significant metabolomic differences among the fermented broths of different jujube varieties, providing an important research foundation for the subsequent secondary analysis and identification of key differential metabolites.

#### Volcano Plots of Differential Metabolites in Different Groups

**Figure 4:**
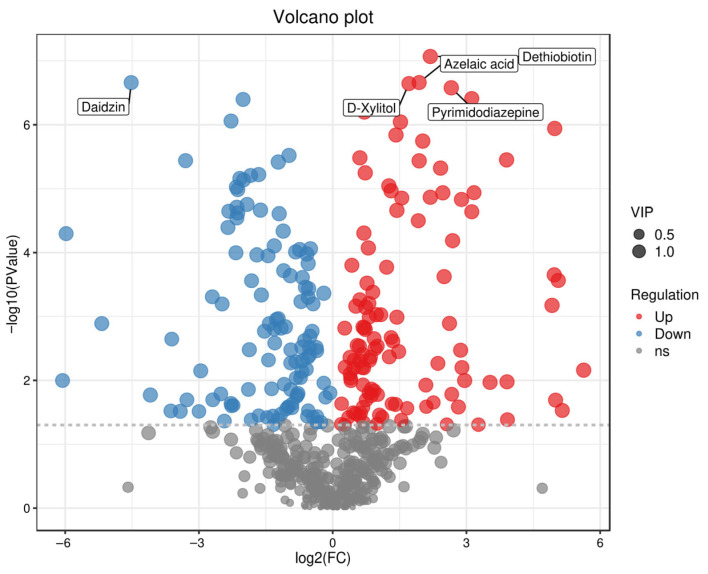
Volcano Plot of Differential Metabolites for One vs. Three. Note: Red indicates up-regulated metabolites, blue indicates down-regulated metabolites (VIP > 1 and *p* < 0.05).

**Figure 5:**
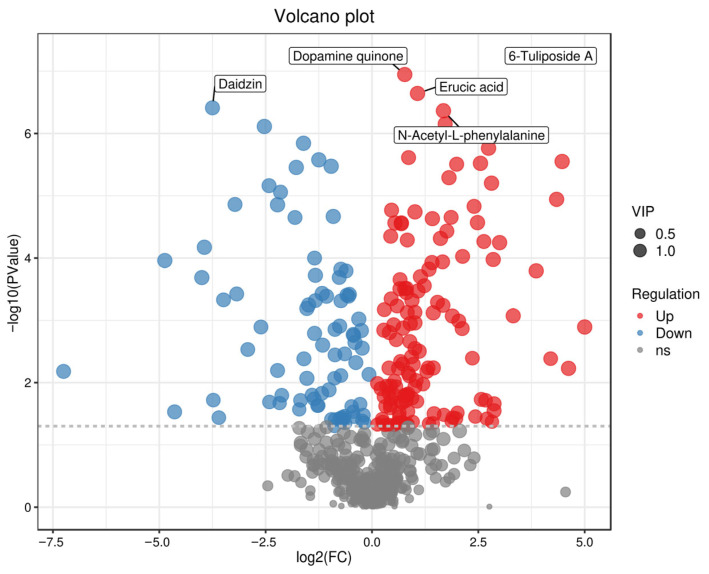
Volcano Plot of Differential Metabolites for One vs. Two. Note: Red indicates up-regulated metabolites, blue indicates down-regulated metabolites (VIP > 1 and *p* < 0.05).

**Figure 6:**
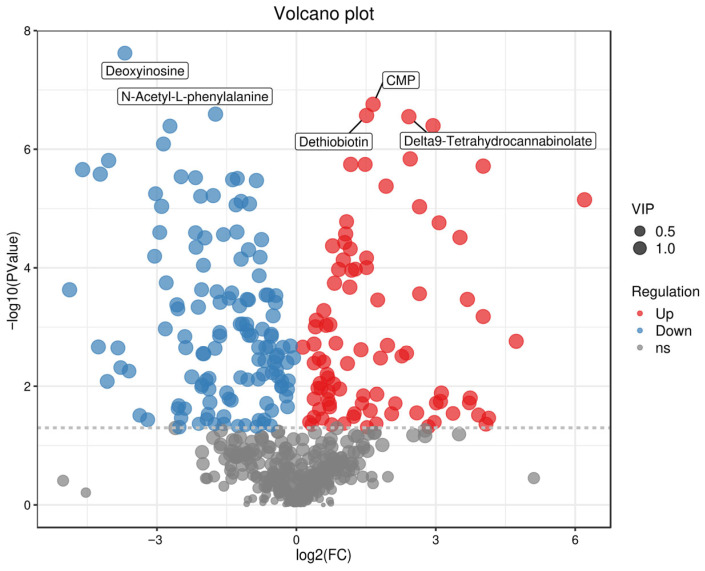
Volcano Plot of Differential Metabolites for Two vs. Three. Note: Red indicates up-regulated metabolites, blue indicates down-regulated metabolites (VIP > 1 and *p* < 0.05).

Volcano plots can intuitively display the distribution of differential metabolites between two sample groups, with red dots representing up-regulated metabolites and blue dots denoting down-regulated metabolites [39,40]. Volcano plot analysis was performed for the three comparison groups (one vs. three, one vs. two, two vs. three), and the top three key differential metabolites in each group were identified. In Figure 4 (one vs. three), a comparison of the fermented broths of Tan jujube (one) and Ban jujube (three) revealed that the top three key differential metabolites were Daidzin, D-Xylitol, and Azelaic acid. In Figure 5 (one vs. two), for the comparison of the fermented broths of Tan jujube (one) and Jun jujube (two), the top three key differential metabolites were Daidzin, Dopamine quinone, and Erucic acid. In Figure 6 (two vs. three), the top three key differential metabolites identified from the comparison of the fermented broths of Jun jujube (two) and Ban jujube (three) were Deoxyinosine, N-Acetyl-L-phenylalanine, and Dethiobiotin. The key differential metabolites among all groups were mainly involved in primary metabolites such as functional carbohydrates, flavonoids, catecholamines, and vitamins.

Fermented jujube broths are rich in D-Xylitol, a functional carbohydrate that plays a crucial role in enhancing immune function and maintaining the balance of intestinal flora [41]. Given the rising prevalence of metabolic syndrome, functional carbohydrates exhibit excellent efficacy in blood glucose regulation [42], and improved utilization of such substances represents a new opportunity for the development of functional fermented jujube products. Isoflavones possess significant biological activities, including inhibiting cancer cell proliferation, exerting antioxidant effects [43], and promoting the growth of normal somatic cells. The high isoflavone content in fermented jujube broths renders them a valuable food source for boosting immune function [44]. Dethiobiotin is a precursor and structural analog of biotin (vitamin B7) with the molecular formula C_10_H_18_N_2_O_3_. Unlike biotin, it lacks a sulfur atom in its structure but serves as a key intermediate in the microbial biosynthesis pathway of biotin [45,46]. The detection of dethiobiotin in fermented jujube broths suggests active microbial metabolic activity in synthesizing essential vitamins.

### 3.3. Multidimensional Screening and Visualization of Differential Metabolites

**Figure 7:**
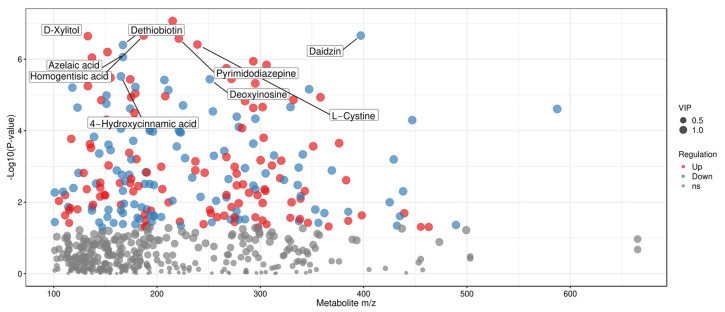
Scatter Plot of Mass-to-Charge Ratio and *p*-value for Differential Metabolites in One vs. Three.

**Figure 8:**
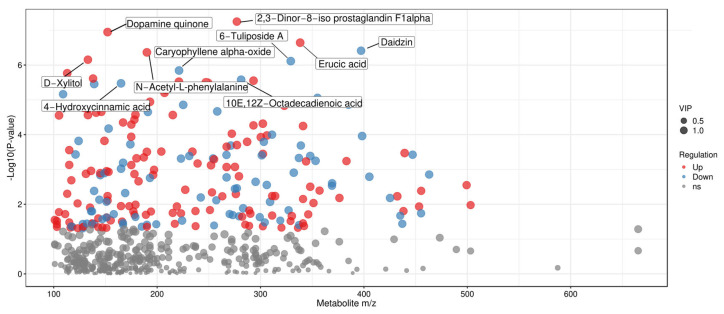
Scatter Plot of Mass-to-Charge Ratio and *p*-value for Differential Metabolites in One vs. Two.

**Figure 9:**
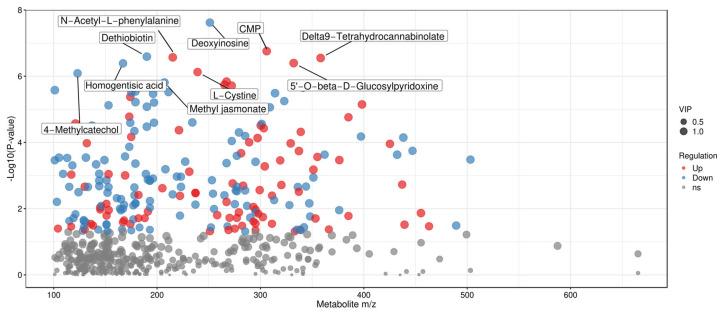
Scatter Plot of Mass-to-Charge Ratio and *p*-value for Differential Metabolites in Two vs. Three. Note: The detection of Delta9-Tetrahydrocannabinolate is an unexpected finding, and its biological origin is still under investigation.

To more intuitively display and screen differential metabolites from the perspective of chemical structure, scatter plots of metabolite mass-to-charge ratio (m/z) versus *p*-value were generated (Figure 7, Figure 8 and Figure 9). Each dot in the plots represents a single metabolite, with the abscissa denoting its m/z value and the ordinate representing the −log10-transformed *p*-value (i.e., −log10 (*p*-value)). The size of each dot corresponds to its VIP value, with larger dots indicating more significant intergroup differences in the corresponding metabolite [47]. These plots enable the clear identification of metabolites with statistically significant differences.

A comparison of Tan jujube (one) and Ban jujube (three) fermented broths revealed multiple dots with significant differences. For example, the dots corresponding to D-Xylitol, Dethiobiotin, Azelaic acid, Daidzin, and other metabolites were located in the upper part of the plot, indicating their extremely low *p*-values and thus highly significant differences.

For the comparison of Tan jujube (one) and Jun jujube (two) fermented broths, metabolites including Dopamine quinone, Erucic acid, and N-Acetyl-L-phenylalanine exhibited significant intergroup differences.

In Figure 9 (two vs. three), the comparison of Jun jujube (two) and Ban jujube (three) fermented broths showed obvious differences in metabolites such as Deoxyinosine, CMP (cytidine monophosphate).

### 3.4. Biological Functions and Mechanisms of Action of Key Differential Metabolites

Comprehensive analysis of the three comparison groups revealed that the key differential metabolites in the three lactic acid bacteria-fermented jujube broths included organic acids, triterpenoid glycosides, alkaloids, flavonoids, functional carbohydrates, vitamins, and sterols [48].

Among them, Linolenic acid was identified as a significantly differential metabolite in two sets of comparisons. As an important polyunsaturated fatty acid [49], linolenic acid exerts extremely vital physiological functions. Studies have shown that, as an essential fatty acid for humans, the physiological functions of linolenic acid are mainly mediated by its metabolites, namely Prostaglandins and Leukotrienes [50]. These metabolites act as important signaling molecules in the body and can regulate various physiological processes such as inflammatory responses, immune reactions, and vascular dilation and contraction. Therefore, linolenic acid plays a pivotal role in anti-aging, anti-inflammation, anti-allergy, blood pressure reduction, visual acuity improvement, blood lipid lowering, as well as the promotion of neural function and brain development [51]. In addition, flavonoids also exhibit significant biological activities. As a class of polyphenolic compounds widely distributed in plants, the antioxidant, anti-inflammatory, and blood circulation-improving effects of flavonoids have been extensively studied. Their mechanisms of action are mainly associated with scavenging free radicals, inhibiting oxidative stress, regulating cellular signaling pathways (e.g., the NF-κB pathway), and improving vascular endothelial function [52,53,54]. Triterpenoids are a class of natural products with diverse pharmacological activities, and their anti-cancer, antibacterial, and antiviral effects have been reported in numerous studies. Their mechanisms of action may involve inhibiting cancer cell proliferation, inducing cell apoptosis, interfering with the viral replication cycle, and regulating the host immune response [55,56,57,58].

### 3.5. Pathway Analysis of Differential Metabolites

#### 3.5.1. Pathway Analysis of Differential Metabolites Based on KEGG Pathway Enrichment

**Figure 10:**
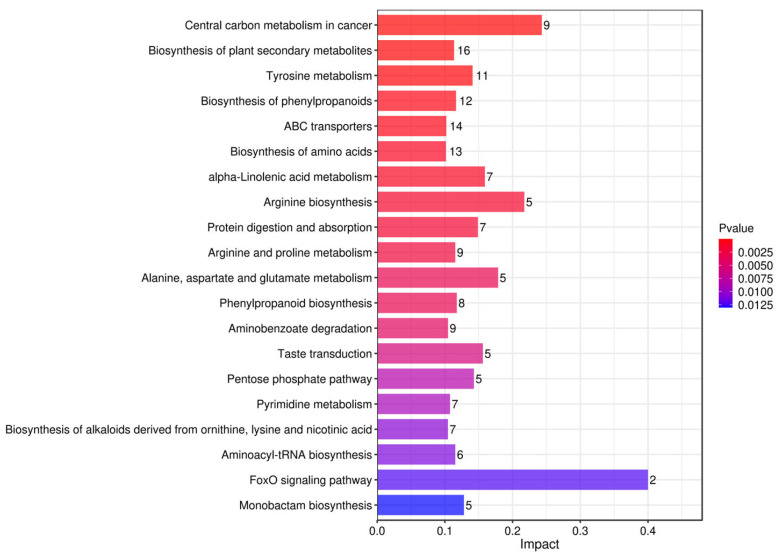
Bar Plot of Influencing Factor Enrichment in Metabolic Pathways for One vs. Three.

**Figure 11:**
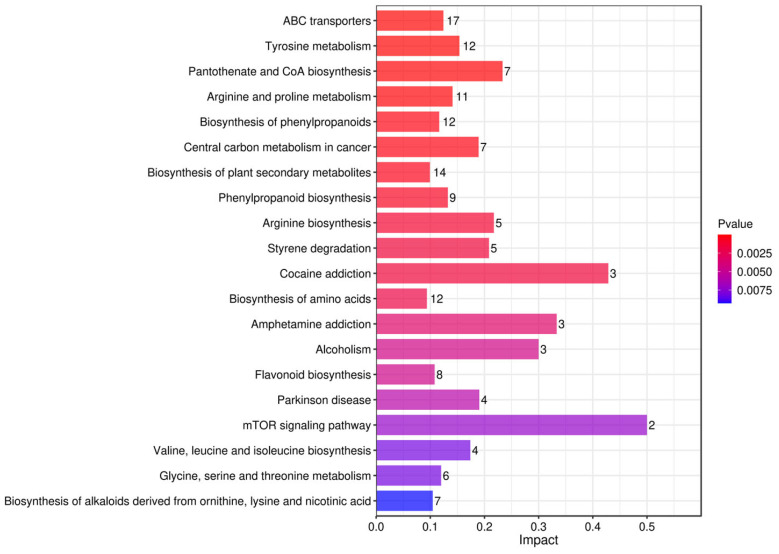
Bar Plot of Influencing Factor Enrichment in Metabolic Pathways for One vs. Two.

**Figure 12:**
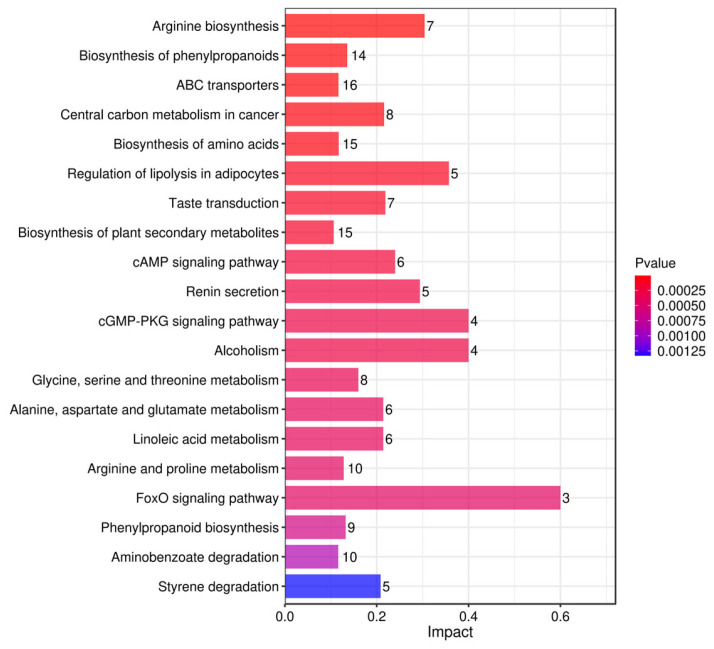
Bar Plot of Influencing Factor Enrichment in Metabolic Pathways for Two vs. Three.

To further investigate the biological processes involved in differential metabolites, we performed a systematic analysis of the metabolites using the KEGG pathway enrichment analysis method [59]. Histograms (Figure 10, Figure 11 and Figure 12) were plotted based on the influence factors in metabolic pathways to intuitively display the importance of each pathway. In the histograms, the ordinate represents metabolic pathways, and the abscissa denotes the Impact value (metabolic pathway impact value) of metabolites enriched in different metabolic pathways. This value can be simply understood as the contribution degree, where a higher value indicates a higher contribution of the detected metabolites under the corresponding pathway. The color is correlated with the *p*-value: the redder the color, the smaller the *p*-value; the bluer the color, the larger the *p*-value.

In the comparison of Tan jujube (one) and Ban jujube (three), the FoxO signaling pathway (Impact = 0.6), Central carbon metabolism in cancer (Impact = 0.4), and Arginine biosynthesis (Impact = 0.3) ranked the top three in terms of both Impact value and enrichment significance. As a core regulatory pathway for cellular stress response and energy metabolism, the high contribution degree of the FoxO signaling pathway suggests that there are key differences between the fermented broths of the two varieties in cellular antioxidant capacity, apoptosis regulation, and energy homeostasis maintenance [60,61,62]. The significant enrichment of Central carbon metabolism in cancer reflects the metabolic differentiation of the two varieties in energy supply processes such as glycolysis and the tricarboxylic acid cycle [63,64,65]. Notably, the prominence of the Arginine biosynthesis pathway suggests distinct variations in nitrogen metabolism between the two varieties. This pathway is closely linked to the accumulation of arginine and proline, which are critical for the umami taste and stress tolerance of the fermented product, indicating that Tan jujube and Ban jujube may differ significantly in their potential to develop flavor precursors and bioactive nitrogenous compounds during fermentation [66,67].

In the comparison of Tan jujube (one) and Jun jujube (two), the mTOR signaling pathway (Impact = 0.5), Cocaine addiction (Impact = 0.45), and Amphetamine addiction (Impact = 0.4) were identified as the core differential pathways. The mTOR signaling pathway is a central node for cell growth, nutrient sensing and metabolic reprogramming [68], and its high Impact value indicates significant differences between the fermented broths of the two varieties in lactic acid bacteria-mediated protein synthesis and lipid metabolism regulation [69]. This implies that the two varieties exhibit distinct efficiencies in nutrient utilization and protein turnover during fermentation, which directly impacts the nutritional quality and texture-related metabolites of the final products. The enrichment of Cocaine addiction and Amphetamine addiction pathways essentially reflects the differentiation of the two varieties in the metabolism and signal transduction of neurotransmitters such as dopamine [70], suggesting that the fermentation process has a variety-specific effect on the accumulation of neuromodulation-related metabolites in jujube juice. This indicates that Jun jujube and Tan jujube may differ in their ability to produce bioactive compounds that influence the nervous system, potentially offering different functional benefits.

In the comparison of Jun jujube (two) and Ban jujube (three), the FoxO signaling pathway (Impact = 0.6), cGMP-PKG signaling pathway (Impact = 0.4), and Alcoholism (Impact = 0.4) exhibited the highest contribution degrees. The repeated high enrichment of the FoxO signaling pathway verifies its core regulatory role in the fermentative metabolic differences among different jujube varieties. This consistent activation suggests that oxidative stress response is a common but variably intense feature across cultivars, likely linked to their distinct polyphenol profiles. As a key pathway for vasodilation and energy metabolism [71], the significant differences in the cGMP-PKG signaling pathway suggest variations between the two varieties in the synthesis of active substances such as nitric oxide and the regulation of energy homeostasis [72]. The enrichment of the Alcoholism pathway is associated with the oxidative stress response of metabolites, which reflects the specific differentiation of the two varieties of fermented broths at the level of antioxidant metabolism [73,74]. Collectively, these pathways highlight that the metabolic divergence between Jun and Ban jujube is largely characterized by their differential strategies in managing oxidative stress and energy regulation, which are crucial for the stability and shelf-life of the fermented functional beverages.

#### 3.5.2. Functional Analysis of Key Metabolic Pathways

Comprehensive analysis of the fermented broths from the three jujube varieties identified six key metabolic pathways, namely central carbon metabolism, plant secondary metabolism, ABC transporter metabolism, tyrosine metabolism, arginine biosynthesis, and phenylpropanoid biosynthesis. These selected pathways exhibited highly significant enrichment (*p*-value < 0.05) in at least two sets of intervarietal comparisons, while meeting the quantitative criterion of no less than 3 differential metabolites enriched per pathway. The six pathways comprehensively cover the critical metabolic processes during jujube fermentation, including energy metabolism (central carbon metabolism), bioactive substance synthesis (plant secondary metabolite biosynthesis, phenylpropanoid biosynthesis), transmembrane substance transport (ABC transporters), and amino acid metabolism (tyrosine metabolism, arginine biosynthesis). All key pathways were annotated to the characteristic differential metabolites identified in the aforementioned analysis—for instance, the arginine biosynthesis pathway was associated with D-Xylitol, and the phenylpropanoid biosynthesis pathway with Daidzin. Moreover, these metabolites showed significant differences in their Z values (Figure 10, Figure 11 and Figure 12), with the pathway functions being highly consistent with the biological effects of the metabolites. Meanwhile, the pathways were correlated with the core mechanisms of lactic acid bacteria fermentation, including lactic acid bacteria-mediated carbon source utilization, secondary metabolite transformation, and nutrient transport [75], which aligns with the nutritional characteristics of jujubes, which are rich in flavonoids and amino acids, thus possessing distinct physiological significance and research value [76].

Among these pathways, central carbon metabolism acts as the core energy hub for lactic acid bacteria’s fermentation of jujubes. Glucose and fructose in jujubes are catabolized via glycolysis and the tricarboxylic acid cycle to supply energy, and also provide carbon skeletons for the proliferation of lactic acid bacteria and the synthesis of organic acids and functional peptides. Intervarietal differences in carbon source content and lactic acid bacteria utilization preferences directly affect the fermentation rate and the accumulation efficiency of functional components [64,65]. Lactic acid bacteria catalyze the structural modification of precursor substances such as flavonoid glycosides in jujubes through esterases and glycosidases [77], and the plant secondary metabolite biosynthesis pathway serves as the major synthetic route for antioxidant and anti-inflammatory bioactive substances in the fermented broths [78,79]. Intervarietal differences in precursor reserves and the interaction with bacterial enzyme systems determine the upper limit of the functional value of fermented products. ABC transporters mediate the uptake of nutrients and the secretion of metabolites in jujube juice [80], and also facilitate the transmembrane accumulation of hydrophobic bioactive substances such as flavonoids and vitamins [81]. Intervarietal differences in nutrient components induce the differential expression of transporter genes, which directly impacts the final content of target functional components [82]. Tyrosine in jujubes is metabolized by lactic acid bacteria to generate nitrogen-containing bioactive compounds such as dopamine and phenolic acid derivatives [11], which are important sources of the neuromodulatory and anti-inflammatory functions of the fermented broths. Intervarietal differences in tyrosine content and bacterial decarboxylase activity [83,84] lead to the differentiation of functional specificities. Lactic acid bacteria synthesize arginine using precursors such as glutamic acid in jujube juice, which is further metabolized to produce nitric oxide and polyamines—this constitutes the core pathway underlying the immunoregulatory function of the fermented broths. Intervarietal differences in nitrogen source reserves and the expression of synthetic enzyme systems directly affect immunoregulatory activity [70,85]. Lactic acid bacteria promote the conversion of phenylalanine in jujubes to phenolic acids such as cinnamic acid and coumaric acid by regulating the activity of phenylalanine ammonia-lyase [86], which not only enhances the antioxidant and antibacterial activities of the fermented broths but also improves microbial homeostasis [87]. Intervarietal differences in conversion efficiency determine the functional advantages of the respective fermented products. Collectively, the integration of pathway analysis with metabolite dynamics reveals that the intervarietal differentiation is not random but functionally directed. Specifically, the upregulation of phenylpropanoid biosynthesis in certain varieties acts as a key determinant of the product’s antioxidant capacity, while the activity of arginine and tyrosine metabolism pathways serves as a biomarker for immunoregulatory potential and flavor profile development. This qualitative mapping between key metabolites and their biological effects bridges the gap between metabolic biochemistry and the physiological properties of the fermented jujube broths, providing a theoretical basis for selecting cultivars with targeted functional characteristics.

## 4. Discussion

In this study, an untargeted metabolomics approach based on liquid chromatography–mass spectrometry (LC-MS) was employed to conduct pairwise comparisons of metabolic characteristics among three jujube varieties (Tan jujube, Jun jujube, and Ban jujube) under the same fermentation system. This research systematically elucidated the specific regulatory rules of substrate varieties on the formation of lactic acid bacteria fermentation products, providing a novel scientific perspective for the precise development of high-value-added fermented jujube products.

A total of 570 metabolites were identified in the fermented broths of the three jujube varieties, with significant intergroup differences and good intragroup repeatability, which verified the reliability of the experimental design and the scientificity of the data. Notably, the characteristic differential metabolites of fermented broths from different varieties exhibited distinct function-oriented features: the high enrichment of linolenic acid in Tan jujube fermented broth was directly associated with its potential functional advantages in anti-inflammation and immune regulation [49,50], providing a material target for the development of targeted anti-inflammatory fermented products; the specific accumulation of D-Xylitol in Ban jujube fermented broth was consistent with its physiological functions in intestinal flora regulation and blood glucose homeostasis maintenance [41,42], offering a clear direction for the targeted development of functional beverages [88]; the differences in the contents of dethiobiotin and Sadenosylmethionine between Jun jujube and Ban jujube fermented broths endued the two varieties with unique values in metabolic regulation and physiological activity modulation [45,46]. Furthermore, these findings suggest that optimizing fermentation strategies—such as selecting specific cultivars rich in precursors or adjusting fermentation time to maximize the release of these bioactives—could significantly enhance the functional value of the final products. Comparative analysis with studies on other shrubs reveals distinct metabolic profiles. Unlike the phenolic-dominated profiles often reported in fermented blueberry or sea buckthorn [89], the jujube broths here showed specific enrichment of D-xylitol and dethiobiotin, highlighting a unique carbon and nitrogen metabolic potential of jujube substrates.

KEGG pathway enrichment analysis further clarified the origin of varietal differences at the mechanistic level, and the enrichment characteristics of six key metabolic pathways profoundly reflected the variety-specific interactions between lactic acid bacteria and jujube substrates. As the energy core of the fermentation process [64], the activity differences in central carbon metabolism directly stemmed from the carbohydrate reserves of different jujube varieties and the carbon source utilization preferences of lactic acid bacteria, which in turn regulated the fermentation rate and the synthesis efficiency of organic acids and functional peptides, serving as the core driver of the differentiation of fermentation characteristics among varieties [65]; the differential expression of the ABC transporter pathway became a key regulatory link for the differences in the accumulation efficiency of functional components by affecting nutrient uptake and metabolite secretion, and the high content of dethiobiotin in Ban jujube fermented broth was directly related to the transport efficiency of vitamin-like substances via this pathway [80,81]; the activity differentiation of the plant secondary metabolite biosynthesis [78,79] and phenylpropanoid biosynthesis pathways [86,87] not only explained the varietal differences in the antioxidant and anti-inflammatory activities of fermented broths, but also highlighted the interaction specificity between lactic acid bacteria enzyme systems (e.g., glycosidases, phenylalanine ammonia-lyase) and jujube secondary metabolic precursors, providing mechanistic support for screening fermented varieties with high antioxidant activity. Of particular note, the FoxO signaling pathway exhibited the highest Impact value in both the comparisons of Tan jujube vs. Ban jujube and Jun jujube vs. Ban jujube, suggesting that this pathway may act as a core hub regulating the metabolic differences in jujube fermentation. These findings align with reports on hawthorn and goji berry fermentation where substrate–enzyme interactions drive metabolic divergence [90]. However, the unique high Impact value of the FoxO pathway in jujube suggests a potentially superior capacity for maintaining energy homeostasis compared to other high-acid fruits [91].

Existing studies on the fermentation of multi-stemmed fruit shrubs, such as sea buckthorn and goji berry, have largely focused on the changes in specific bioactive components (e.g., flavonoids or vitamins) and their antioxidant capacities [92]. While these studies confirmed that lactic acid bacteria fermentation can increase the content of bioactive substances in plant raw materials through enzymatic reactions [93,94,95], they often lacked a systematic elucidation of the metabolic network response. In addition, previous studies have mostly focused on the fermentation characteristics of a single jujube variety [16,17]. Through the pairwise comparison design, this study for the first time systematically constructed the fermentation metabolic fingerprint of three main jujube varieties, overcame the limitations of traditional studies, and provided a more comprehensive reference for the analysis of variety-specific fermentation mechanisms.

The innovative value of this study lies in clarifying the core differential targets and key regulatory pathways of fermentation metabolism in different jujube varieties, and establishing an association network of “variety–metabolite–pathway–function”, which provides directly applicable scientific evidence for the precise selection of fermentation raw materials and the targeted development of functional products. However, the study still has certain limitations: the transformation contribution of a single strain in the composite lactic acid bacteria to jujube metabolites has not been clarified, and the biological activities of differential metabolites are only inferred based on existing literature, lacking verification by in vitro cell experiments and animal models. A key limitation is the lack of functional validation, which weakens the claimed impact of our findings. While in vitro antioxidant assays such as ORAC, FRAP, and ABTS could have been employed to provide direct functional evidence for the identified metabolites, these experiments were beyond the scope of the current study.

Future research can focus on the following three aspects: first, analyze the interaction mechanism between each strain in the composite lactic acid bacteria and different jujube varieties, and identify key functional strains and their core metabolic enzyme systems; second, construct a comprehensive “metabolite–functional effect” network by integrating the identified differential metabolites with functional activity assays (e.g., antioxidant, anti-inflammatory, and gut microbiota-modulating evaluations). Specifically, statistical correlation analyses, such as Pearson correlation or network modeling, will be employed to link individual metabolite abundances to specific biological effects, allowing for the identification of key metabolites that directly contribute to the observed bioactivities. This strategy will transform metabolomic findings into concrete functional evidence. Third, optimize fermentation process parameters (e.g., inoculum size, fermentation time) based on the key pathways and differential metabolites identified in this study to realize the targeted preparation of fermented jujube products with high functional value. In addition, expanding the research on the effects of different processing and pretreatment methods on jujube fermentation metabolism can provide more comprehensive technical support for the diversified and high-quality development of the jujube fermentation industry. Collectively, these future directions will facilitate the precise development of high-value functional foods from *Ziziphus jujuba*, bridging the gap between metabolic mechanisms and industrial applications.

## Data Availability

The original contributions presented in the study are included in the article; further inquiries can be directed to the corresponding author.
